# The Essential Oil of Tunisian Halophyte *Lobularia maritima*: A Natural Food Preservative Agent of Ground Beef Meat

**DOI:** 10.3390/life12101571

**Published:** 2022-10-10

**Authors:** Boutheina Ben Akacha, Jaroslava Švarc-Gajić, Khaoula Elhadef, Rania Ben Saad, Faiçal Brini, Wissem Mnif, Slim Smaoui, Anis Ben Hsouna

**Affiliations:** 1 Laboratory of Biotechnology and Plant Improvement, Centre of Biotechnology of Sfax, P.O. Box 1177, Sfax 3018, Tunisia; 2 Faculty of Technology, University of Novi Sad, Bulevar Cara Lazara 1, 21000 Novi Sad, Serbia; 3 Laboratory of Microbial, Enzymatic Biotechnology and Biomolecules (LBMEB), Center of Biotechnology of Sfax, University of Sfax, Road of Sidi Mansour Km 6, P.O. Box 1177, Sfax 3018, Tunisia; 4 Department of Chemistry, Faculty of Sciences and Arts in Balgarn, University of Bisha, P.O. Box 199, Bisha 61922, Saudi Arabia; 5 ISBST, BVBGR-LR11ES31, Biotechpole Sidi Thabet, University of Manouba, Ariana 2020, Tunisia; 6 Departement of Enviromental Sciences and Nutrition, Higher Institute of Applied Sciences and Technology of Mahdia, University of Monastir, Monastir 5000, Tunisia

**Keywords:** *Lobularia maritima* essential oil, antimicrobial properties, minced meat preservation, quality assessment

## Abstract

This study was directed towards the investigation of the chemical composition and antimicrobial properties of the essential oil of Tunisian halophyte *Lobularia maritime* (*LmEO*). The antibacterial effects against major food-borne pathogenic and food spoilage bacteria were tested using the well diffusion method, followed by the determination of the minimum inhibitory (MIC) and bactericidal (MBC) concentrations. The essential oil has shown strong antimicrobial activity against eight pathogenic strains, which was attributed mostly to predominant constituents of the essential oil: benzyl alcohol, linalool, terpien-4-ol and globulol, as well as to synergistic effects of its major and minor constituents. Considering strong antimicrobial effects of the tested essential oil, it was further tested as a natural alternative to food preservatives, using minced beef meat as a model system. Minced beef meat was spiked with 0.019, 0.038, and 0.076% of the essential oil and stored during 14 days at 4 °C, monitoring its microbiological, physicochemical, and sensory properties. Chemical analyses revealed that meat treated with 0.076% of *LmEO* at underwent a significant decrease (*p <* 0.05) in primary and secondary lipid oxidation and reduced metmyoglobin accumulation compared with control samples. Furthermore, microflora proliferation in the meat model system spiked with 0.076% of *LmEO* was significantly (*p <* 0.05) reduced in comparison to control. In addition, two multivariate exploratory techniques, namely principal component analysis (PCA) and hierarchical analysis (HCA), were applied to the obtained data sets to describe the relationship between the main characteristics of the meat samples with and without essential oil addition. The chemometric approach highlighted the relationships between meat quality parameters. Overall, results indicated that the essential oil of *Lobularia maritima* deserves to be considered as a natural preservative in the meat industry.

## 1. Introduction

Meat represents one of the most perishable foodstuffs [1]. In addition to lipid oxidation and enzymatic reactions, meat spoilage is almost always caused by microbial growth. The breakdown of fats, proteins, and carbohydrates in meat leads to the development of off-flavours, compromising the product quality. To control and eliminate foodborne bacteria and extend the shelf life of meat and meat products, the most commonly applied are synthetic preservatives [2]. However, these have been restricted in several countries, due to their undesirable long-term toxicological effects, including carcinogenicity [3]. The current tendencies in consumers are oriented toward the use of more natural, healthy, and “organic” food, prompting the research, development, and application of new natural products with antimicrobial and antioxidant activities as alternatives to synthetic preservative [4].

Essential oils have been used for thousands of years for food preservation and to provide characteristic flavours for certain foods and beverages [4]. Many essential oils exhibit antimicrobial activity and may inhibit the growth of pathogenic microorganisms [5], thereby improving food safety. Many publications have confirmed the possibility of using essential oils, such as oregano, rosemary, sage, and thyme oil, in meats, to extend the shelf life [6,7]. Many plants, especially those used as spices and condiments, synthesize antimicrobial compounds as a natural defence mechanism [8], for which reason it was interesting to study in this respect the *Lobularia maritima,* the saline halophyte commonly found on the Tunisian coast, which hasn‘t been studied sufficiently for real applications, according to our knowledge.

This work was thus, primarily oriented towards the study of the antimicrobial and preservation potentials of the *Lobularia maritima* L*. Desv* (Brassicaceae) essential oil, and potential uses in the food sector. The plant is tolerable to high concentrations of salt, even up to 400 mM, and is commonly used for ornamental purposes [9]. Available studies related to the bioactivity of this plant were mostly focused on plant extracts, rather than essential oils. Marelli et al. [10] reported several important health benefits of the extracts of aerial *Lobularia maritima* (L.) *Desv.* parts. With respect to essential oil analysis, insecticidal activity against several grain pests (*Sitophilus oryzae, Tribolium castaneum, Callosobruchus maculatus*) [11]. They are also exploited as additives or natural ingredients in foods that claim health benefits) [5], as well as hepatoprotective properties [12], have been previously reported for *Lobularia maritima*. Antimicrobial activity of the *Lobularia maritima* L. Desv essential oil against common foodborne pathogens and food spoilage bacteria was tested in vitro, followed by the study of the potential application for ground beef preservation and amelioration of physicochemical, microbiological, and sensory properties. Models correlating antioxidant, antimicrobial, and sensory properties of beef samples spiked with *L. maritima* essential oils were defined by applying chemometric PCA and HCA approaches.

## 2. Materials and Methods

### 2.1. Collection of the Plant Material

*Lobularia maritima* plant was collected in April 2017 in the region of Chebba (Mahdia, North: 35.14° and East: 11.07°). The plant was identified and authenticated by Prof. Mohamed Chaieb (Department of Botany, Faculty of Science, University of Sfax, Tunisia), according to *the Flora of Tunisia* [13]. After harvesting, the aerial parts were dried in the dark at room temperature for 2 days.

### 2.2. Chemicals

Anhydrous sodium sulphate, benzyl alcohol, linalool, terpien-4-ol, globulol, dimethyl sulfoxide (DMSO), butylated hydroxytoluene (BHT), streptomycin, thiazolyl blue tetrazolium bromide (MTT), potassium phosphate buffer (K_3_PO_4_) were purchased from Sigma-Aldrich Co. (St. Louis, MO, USA). Muller–Hinton agar was purchased from Bio-Rad(Steenvoorde, France). Agar plates of MH agar and red-violet bile glucose medium were attained from Oxoid Ltd., Basingstoke, UK.

### 2.3. Lobularia Maritima Essential Oil (LmEO) Extraction

The essential oil was obtained from 1.2 kg of aerial parts previously ground and mixed with 4 L of distilled water. Hydrodistillation was performed for 4 h using a Clevenger-type apparatus [14]. The aqueous phase was extracted with hexane (6 × 50 mL) and dried with anhydrous sodium sulphate for yield determination. After filtration, the solvent was removed by distillation under reduced pressure in a rotary evaporator, and the pure oil was stored at 4 °C in the dark until analysis began. The pure oil was stored at 4 °C in the dark. The yield of separated essential oil was calculated as [14]:

Oil (%*v/w*)  =  volume (mL)/weight of sample (g) × 100

### 2.4. Gas Chromatography Mass Spectrometry

The analysis of extracted essential oils was carried out by gas chromatography-mass spectrometry (GC MS using a Shimadzu-QP2010SE 15A operating at 70 eV ionization energy, equipped with an Rtx-5MS (phenyl methyl siloxane 30 m × 0.25 mm, 0.25 μm film thicknesses). As a carrier gas, helium was used with a flow rate of 0.9 mL/min and a split ratio of 1:20. The compounds were identified by the comparison of calculated Kovats retention index with those previously reported in the literature and by comparison of their mass spectra with the Wiley and NIST libraries or mass spectra previously reported in the literature [15].

### 2.5. Antimicrobial Activity

Authentic pure cultures of bacteria were obtained from the international culture collections: American type culture collection (ATCC) and the local culture collection of the Centre of Biotechnology of Sfax, Tunisia. They included Gram-positive bacteria: *Bacillus cereus ATCC 14579, Staphylococcus aureus ATCC 25923, Enterococcus faecalis ATCC 29212*, *Micrococcus luteus ATCC 1880* and *Listeria monocytogenes ATCC 19117* and Gram-negative bacteria: *Salmonella enterica ATCC 43972, Escherichia coli ATCC 25922* and *Pseudomonas aeruginosa ATCC 9027*. Bacteria were cultivated in Muller–Hinton agar (MH) at 37 °C for 12–24 h except for *Bacillus* species which were incubated at 30 °C.

#### 2.5.1. Agar Diffusion Method

Antibacterial tests were performed by agar well diffusion method as described by [16], and broth micro-dilution assay using sterile Mueller–Hinton (MH) media (Bio171 Rad, France) for bacterial strains. Working cell suspensions were prepared and 100 μL were evenly spread onto the surface of the agar plates of MH agar (Oxoid Ltd., Basingstoke, UK). The wells were carved in the agar using a sterile Pasteur pipette (6 mm in diameter). The *LmEO* were dissolved in dimethyl sulfoxide (DMSO) solution. Thus, 60 μL were placed into the wells and the plates were incubated at 37 °C for 24 h. Streptomycin (50 μg/wells) served as a positive, and DMSO served as a negative control. Antimicrobial activity was assessed by measuring the diameter of circular inhibition zones within the well diameter. Tests were performed in triplicate.

#### 2.5.2. Determination of MIC and MBC

Minimum inhibitory concentrations (MICs) of *LmEO* were calculated based on the broth microdilution method in 96 microplates [16] against a panel of 8 microorganisms representing different species of different ecosystems. Ten microliters of cell suspension were added to each test well. MIC (%) values were assessed as the lowest *LmEO* concentration that inhibited the visible growth of each tested bacterium [5]. As an indicator of microorganism growth, 25 μL of thiazolyl blue tetrazolium bromide (0.5 mg/mL) (Sigma-Aldrich, Taufkirchen, Germany) was added to the wells and incubated for 30 min. The colourless tetrazolium salt acts as an electron acceptor and was reduced to a red-coloured formazan product by biologically-active organisms.

The minimum bactericidal concentration (MBC) was defined as the lowest concentration at which 99% of the bacteria were killed. It was determined by removing 10 μL from each well and inoculating in MH plates in strings. After 24 h of incubation at 37 °C, the number of surviving organisms was determined. Available standards benzyl alcohol, linalool, terpien-4-ol and globulol were also tested under identical conditions to compare their activities with those of the studies essential oil.

### 2.6. Preparation of Minced Beef Meat

Fresh post-rigorous lean beef muscle was obtained in a slaughterhouse in Sfax-Tunisia and transported to the laboratory in polystyrene cans isolated on ice within an hour of the cutting process. The pieces of meat prepared as described above were minced in a sterile grinder and was divided into five lots: lot 1 and lot 2 were used as controls, lot 2: 0.01% of a chemical antioxidant butylated hydroxytoluene (BHT) was added, while *LmEO* was added to other three lots at 0.019% (*1LmEO*), 0.038% (*2LmEO*) and 0.076% (*3LmEO*). A homogeneous mixture of each sample (three replicates) was kept under a vacuum using plastic bags. Finally, all samples were stored for 14 days at 4 °C, and quality parameters were analyzed on days 0, 3, 7, 10, and 14.

### 2.7. Quality Control

#### 2.7.1. Microbiological Analysis

Aerobic plate counts (APC), aerobic psychrotrophic (PTC), and *Enterobacteriaceae* counts were evaluated as previously described, implementing meet samples processing according to the ISO 7218 [17]. APC was determined on a plate count agar (PCA, Oxoid, Basingstoke, UK) after incubation at 30 °C for 48 h [18]. PTC was determined as described above for APC, with the exception that plates were incubated at 7 °C for 10 days [19]. The number of enterobacteria was counted on red-violet bile glucose medium (VRBG, Oxoid, Basingstoke, UK) after incubation at 37 °C for 24 h [20].

#### 2.7.2. pH Determination

The pH was determined in homogeneous mixtures of meat with distilled water. Five grams of meat sample were homogenized in 50 mL of distilled water (pH 7.00), and the mixture was filtered [16]. The pH of filtrate was measured using a pH meter (Model: YK-21PH) at each sampling point.

#### 2.7.3. Evaluation of Protein Oxidation in Beef Meat

Protein oxidation was appraised based on the rate of metmyoglobin (MetMb%) formation. The contents of MetMb in ground beef and raw beef patties were determined as described by ben Hsouna et al. [16]. Briefly, 5 g of samples were homogenized in 25 mL of ice-cold 0.04 M potassium phosphate buffer (K_3_PO_4_) (pH 6.8) for 10 s. The homogenate was allowed to stand for 1 h at 4 °C after what it was centrifuged at 4500 g for 30 min at 4 °C. The supernatant was filtered through Whatman No. 1 filter paper and the absorbance was measured at 572, 565, 545, and 525 nm using a Pye-Unicam (Unicam Ltd., Dowlish Ford, UK) spectrophotometer. The percentages of MetMb were calculated based on these absorbance values according to Krzywicki (1982) [21] using the following formula:

MetMb% = [−2.51 (A572/A525) + 0.777 (A565/A525) + 0.8 (A545/A525) + 1.098] × 100

#### 2.7.4. Evaluation of Lipid Oxidation in Beef Meat

Lipid oxidation was evaluated based on the primary and secondary lipid oxidation products determination. Primary lipid oxidation products involved conjugated dienes (CD) and lipid hydroperoxides, whereas malondiadehyde defined secondary lipid oxidation products (TBARS—thiobarbituric acid reactive substances).

CDs were assessed according to Srinivasan et al. [22], and the results were expressed as μmol/mg of meat sample. Meanwhile, the contents of TBARS were determined according to Eymard et al. [23]. TBARS values were expressed as mg of malonaldehyde equivalent per kg of a sample (mg MDA-eq/kg of meat).

#### 2.7.5. Sensory Evaluation

Twenty experienced panellists were selected from the staff at the Sfax Biotechnology Centre to independently assess the colour, appearance, odour, and overall acceptability of the ground meat samples, for each day of storage. Each sample was evaluated in two sessions. Panellists were randomly given coded samples, and sensory attributes were rated using 9-point intensity scales (1 = very poor; 9 = very good).

### 2.8. Statistical Analysis

All measurements were done after 0, 3, 7, 10, and 14 days of meet storage and experiments with five treatments were utilized in a randomized complete block design. Moreover, at each storage time, three replications were performed. Except for sensory analysis, a two-way analysis of variance (ANOVA) was conducted for all variables, and in case of difference, the means were compared by using the Tukey test at 5% significance.

To group the samples according to the number of microbes, the lipid/protein oxidation, and the sensory parameters during the five storage times, all variables were scaled automatically before applying chemometrics. By using the XLSTAT software for Windows (version.2022), PCA and HCA were performed to discriminate between samples. Dendrograms were established to obtain a two-dimensional projection of the similarity or dissimilarity of the samples.

## 3. Results and Discussion

### 3.1. GC MS Analysis of the Essential Oil

The hydrodistillation of *L. maritima* aerial parts yielded 2.4% (*v/w*) of a yellow oil, with a particularly high yield compared to the essential oils yields of same species coastal plants, such as *Crithmum maritimum L.* and *Inula crithmoïdes L.* Reported essential oil contents for these plants were much lower (0.34% and 0.15% (*w/w*), respectively) [24] in comparison to the calculated essential oil yield for *L. maritima* studied in this work. GC MS analysis revealed the presence of 40 constituents, among which 90.55% were, identified (Table 1). The volatile oil contained 74.40% of oxygenated monoterpenes and 24.13% of monoterpene hydrocarbons. The major identified oil constituents were linalool (22.43%), benzyl alcohol (8.65%), 1-phenyl butanone (7.33%), α-cadinol (4.91%), globulol (4.32%), α-terpineol (3.9%), ledol (3.59%), α-pinene (3.51%) and pulegone (3.33%). It is important to note that the oxygenated monoterpenes were present in a relatively high amount (>74.40%).

### 3.2. Antimicrobial Activity

**Figure 1:**
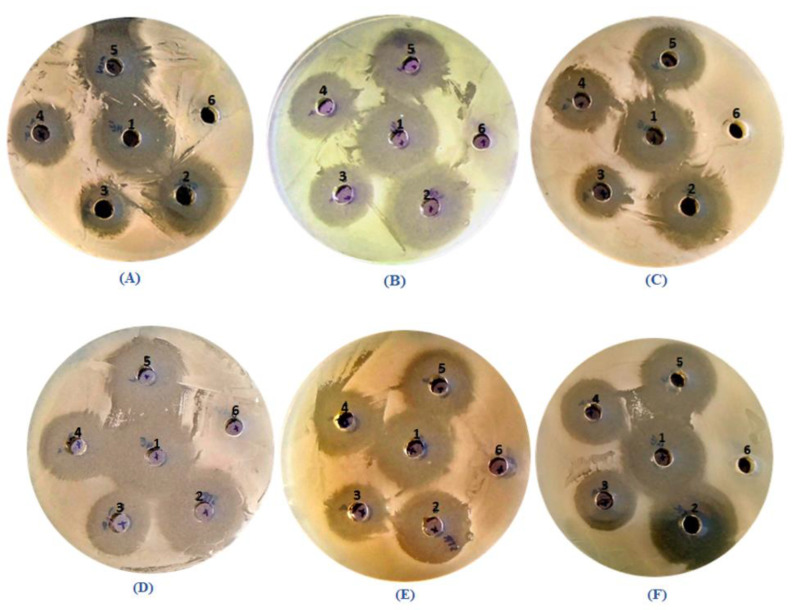
Growth inhibition zones of *LmEO* in different bacterial strains: (**A**) *Bacillus cereus* ATCC 14579; (**B**) *Listeria monocytogenes* ATCC 1911; (**C**) *Enterococccus faecalis* ATCC 29212; (**D**) *Pseudomonas aeruginosa* ATCC 9027; (**E**) *Escherichia coli* ATCC 25922; (**F**) *Salmonella enterica* ATCC 43972. (1): *LmEO* = *Lobularia maritima* essential oil tested in its raw state at 1.8 mg/well; (2): reference antibiotic Streptomycin at 20 μg/well; (3): 1/8 diluted oil at 0.225 mg/well; (4): 1/4 diluted oil at 0.45 mg/well; (5): 1/2 diluted oil at 0.9 mg/well and (6): Negative control used.

**Figure 2:**
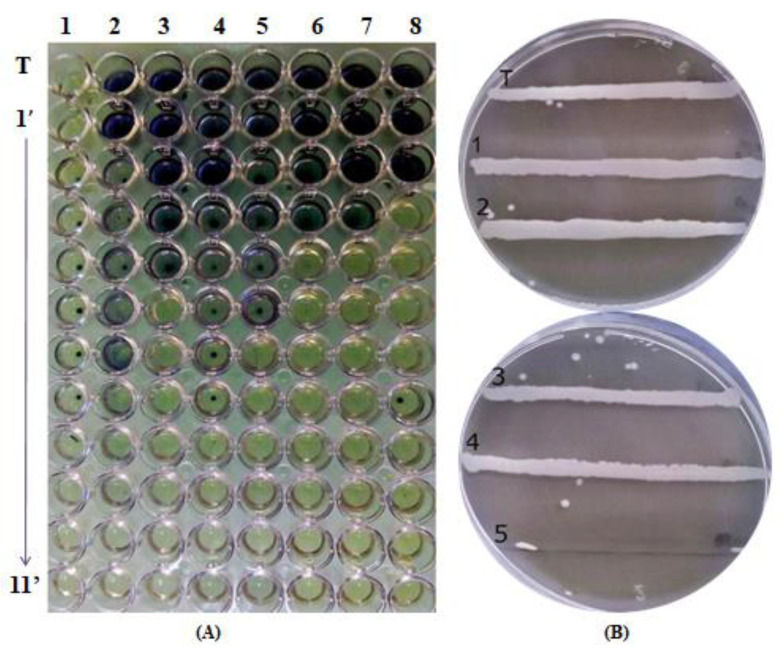
The determination of MIC and MBC in *LmEO*. (**A**) Determination of the MIC by the microdilution method. (1) *Micrococcus luteus*; (2): *Staphylococcus aureus*; (3) *Enterococccus faecalis*; (4) *Escherichia coli*; (5) *Salmonella enterica*; (6) *Listeria monocytogenes;* (7) *Pseudomonas aeruginosa*; (8) *Bacillus cereus*; 1’ to 11’ the different decreasing concentrations from 5000 μg/mL to 4.75 μg/mL and (T) Negative control. (**B**) The determination of BMC determination against *Salmonella enterica* by the streak method; (T) negative control (1) 4.75 μg/mL; (2) 9 μg/mL; (3) 19 μg/mL; (4) 39 μg/mL and (5) 78 μg/mL.

Antimicrobial activity of *LmEO* was assessed by measuring inhibition zones of bacterial growth (mm) (Figure 1), as well as by determining minimum inhibitory concentration (MIC), and minimum bactericidal concentration (MBC) (µg/mL) (Figure 2).

The antibacterial activity of the essential oil was evaluated against Gram+ strains: *Bacillus cereus*, *Staphylococcus aureus*, *Enterococcus faecalis*, *Micrococcus luteus*, and *Listeria monocytogenes,* and against Gram- strains: *Salmonella enterica, Escherichia coli, Psudomonas aeruginosa*. The zones of inhibition varied between 12 and 21 mm for the 1/8 diluted oil as shown in Table 2. Among the Gram+ bacteria tested, the greatest zones of inhibition were detected against *L. monocytogenes* (26.6 mm), *B. cereus* (28.6 mm), and *E. feacalis* (27.3 mm). Among Gram+ strains, the greatest inhibition zones were observed against *S. enterica* with an inhibition zone of 33 mm. The zones of inhibition for the reference antibiotic Streptomycin (20 μg/well), used as a positive control, ranged from 14 to 27.3 mm. The negative control showed no inhibitory effect against the tested bacteria. The microorganisms tested in the present study are among the most important human pathogens known to be opportunistic to humans and animals and to cause food contamination and spoilage [5]. The results obtained are of great importance, especially in the case of *B. cereus* and *S. aureus*, which are well known for their resistance to several phytochemicals, and food production, and for the production of several types of enterotoxins that cause gastroenteritis [25]. Although Hazzit et al. [26] reported that *S. aureus* was resistant to the essential oil of Thymus species, the above results suggest that the essential oil of *Lobularia maritima* is active and can potentially be useful for food preservation.

*LmEO* was found to have significant antibacterial activity against all tested Gram-positive and Gram-negative bacteria, with MIC values of 19–65 µg/mL, and 25–32 µg/mL, respectively. Most studies examining the activity of essential oils of plants against microorganisms responsible for food spoilage show that they are more active against Gram-positive bacteria than Gram-negative [12]. Indeed, Gram-negative bacteria are less sensitive to the action of antibacterial agents due to the presence of the outer membrane surrounding the cell wall, which limits the diffusion of hydrophobic compounds through its lipopolysaccharide layer [27]. Determination of the inhibition zone does not allow a linear correlation between the diameter of the inhibition zone and the antimicrobial concentration. Thus, the diffusion test is qualitative and does not distinguish between bactericidal and bacteriostatic effects. For this reason, an additional broth micro-dilution assay was conducted to quantitatively express the antibacterial potential of the studied oil, through MIC and MBC determination (Table 3).

In the tested concentration range from 19 µg/mL to 65 µg/mL, the essential oil of *L. maritima* exerted bacteriostatic or bactericidal effects on different bacteria. The most sensitive bacteria to the tested essential oil showed to be *Listeria monocytogenes*, *Micrococcus luteus,* and *Pseudomonas aeruginosa*, with very low MICs (Table 3).

Bacteriostatic effects of *L. maritima* essential oil were observed in *S. aureus, E. faecalis, M. luteus, E. coli*, and *L. monocytogenes* as the MIC/CMB ratio for these strains was ≤4. Generally, essential oils rich in oxygenated monoterpenes appear to have much greater antimicrobial activity than oils rich in monoterpene or sesquiterpene hydrocarbons [28]. Thus, the antibiotic activity of *L. maritima* may be attributed to the presence of relatively high proportions of oxygenated monoterpenes. At this stage of the work, it is difficult to precise the compound responsible for the antimicrobial activity of *L. maritima* oil, since it is characterized by a complex mixture of constituents. Nevertheless, the antimicrobial effect could be attributed to the synergistic effects of major and minor compounds identified by GC MS analysis.

Mith et al. [29] tested 15 essential oils against food-borne and food-spoilage bacteria, relating their activities to major essential oil constituents. In general, the study confirmed that essential oils of oregano, cinnamon and thyme exhibited the strongest antimicrobial activities against major food-borne pathogens, with MIC values ≥0.125 µL/mL and MBC values ≥0.25 µL/mL. By relating their antimicrobial activity with the major oil constituents, the authors deduced that oxygenated compounds, like thymol, carvacrol and cinnamaldehyde, abundant in essential oils of oregano, cinnamon and thyme, were responsible for such high antimicrobial activity. Antibacterial activity of the essential oil of *B. dracunculifolia*, a shrub native to Brazil, was also evaluated against food-borne pathogens, but the activity was inferior in comparison to tested essential oil of *L. maritima.* MIC values for this plant, against eight tested bacterial strains, were in the range from 0.50 to 12.65 mg/mL [30].

**Figure 3:**
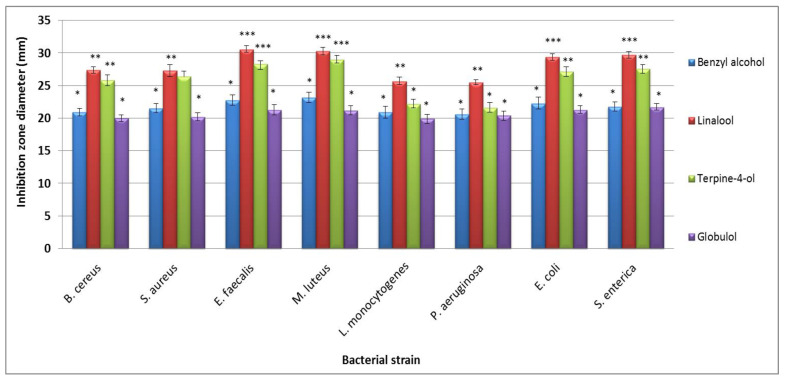
The antibacterial activity of selected standard *LmEO* constituents, expressed as inhibition zones. Diameter of the inhibition zones are given as the mean ± SD of triplicate experiments. * *p <* 0.05 **; *p <* 0.01 and *** *p <* 0.001 indicate significant differences from inhibition zone diameter value.

Selected available standard compounds, identified previously in the essential oil of *L. maritima,* were individually tested for their antibacterial activity under same conditions (Figure 3).

In general, linalool exhibited the greatest inhibition zone against all eight tested bacteria, followed by terpine-4-ol. Tested standard compounds are common constituents of many essential oils and have been proven to be particularly effective against some species of Gram-positive and Gram-negative bacteria [25]. The strong antimicrobial activity of the essential oils against almost all the susceptible microorganisms has been mostly associated with the presence of high concentrations of monoterpenes [31,32,33]. However, the synergistic effect of minor constituents should be taken into consideration in overall antimicrobial activity. In fact, both the synergistic effects and the chemical diversity of major and minor constituents present in the essential oils account for their overall biological activity [5].

### 3.3. Preservation of Raw Minced Beef Meat with LmEO

Considering previously proven antibacterial properties of *LmEO* further study was directed towards investigation of its possible use as a natural preservative in raw ground beef during refrigerated storage at 4 °C. The minced beef meat was spiked with *LmEO* in the following concentrations (*w/w*): 0.019% (*1LmEO*), 0.038% (*2LmEO*), and 0.076% (*3LmEO)*, equivalent to the MIC, 2 × MIC and 4 × MIC values of *LmEO* against *Listeria monocytogenes* strain ATCC 19117, respectively.

#### 3.3.1. Microbiological Evaluation

The aerobic storage of refrigerated meat produces a high redox potential on the surface that favours the growth of bacteria. Changes in the microbial flora of raw ground beef during refrigerated storage are presented in Table 4. At the beginning of the experiments, the minimum level of aerobic plate count (APC) in the control sample of ground beef was about 2.29 ± 0.35 log10 CFU/g of meat. On the 14th day of storage at 4 °C, the control sample exceeded the maximum recommended limit (6.7 log10 CFU/g) [17] with the signs of spoilage, such as a slightly fetid odour, indicating a shelf life of 10 days for untreated meat. However, samples treated with butylated hydroxytoluene (BHT), *1LmEO*, *2LmEO*, and *3LmEO*, showed a delay in the growth of APC for about 1, 1.25, and 1.66 log10 CFU/g extending the shelf life up to 14 days during storage at 4 °C. These results demonstrated that the addition of *LmEO* significantly improved the quality of raw ground beef during refrigerated storage. Moreover, the results of our study were in agreement with those reported by Michalczyk et al. [33]. The authors applied the essential oils of *Coriandrum sativum* and *Hyssopus officinalis* at a concentration of 0.02% to ground beef meat stored at 5 °C and 6 °C. This study reported that treatment with these oils resulted in a slight reduction in the development of APC. The addition of tested essential oils delayed the growth of bacteria for about 1.0 log10 CFU/g compared to the control sample.

In the case of meat products, refrigeration alone may not provide a sufficiently effective barrier to controlling psychotropic pathogenic microorganisms (PTC). The evolution of aerobic psychotropic counts on ground beef during 14 days of storage at 4 °C is shown in Table 4. The initial number of PTC was around 2 log10 CFU/g meat in all samples. During storage at 4 °C, the psychrophilic flora in the control meat sample showed a sharp increase reaching the value of 6.33 log10 CFU/g meat, which corresponded almost to the maximum recommended limit [20]. At this stage, this meat is considered an inconsumable product posing serious sanitary risks for consumers. However, a less accentuated proliferation of PTC was noticed for the samples with *LmEO* addition compared to the two controls. The *2LmEO* and *3LmEO* samples treated with *L. maritima* essential oils at concentrations of 0.038% and 0.076%, respectively, did not reach the maximum PTC until the 14th day of storage at 4 °C. These treatments extended the shelf life of the meats up to 14 days of storage with a delay in PTC growth of approximately 1.16 log10 CFU/g and 1.54 log10 CFU/g, respectively.

The antimicrobial effect of *LmEO* in stored minced beef meat was also studied on *Enterobacteriaceae* (Table 4). The number of *Enterobacteriaceae* at the beginning of the storage period was below 1 log10 CFU/g in all meat samples. During storage, the control sample showed a strong increase of this flora, reaching the total number of *Enterobacteria* of 3.47 log10 CFU/g on day 14th, however, it did not exceed the maximum recommended limit (4.0 log10 CFU/g) [21]. The number of *Enterobacteriaceae* in the meat samples treated with BHT and *L. maritima* essential oil did not exceed the value indicated by the standard during the entire storage period at 4 °C. The addition of 0.076% of the essential oil (*3LmEO*) resulted in a delay in the growth of *Enterobacteriaceae* for about 1.7 log10 CFU/g compared to the control samples. The use of 0.076% of *L. maritima* essential oil appeared to slow the growth rate of *Enterobacteriaceae* with maximum contamination levels on day 10 and day 14 of 1.64 and 1.78 log10 CFU/g, respectively. The contamination levels were significantly lower (*p < 0.05*) compared to the contamination of the control minced meat. Therefore, our findings suggested that the addition of *LmEO* to the minced beef prolongs the shelf life of the product by up to 14 days due to the inhibition of deteriorating microorganisms. Similarly, Hussain et al. [34] studied the effects of the addition of 0.01, 0.025, 0.05, and 0.5% (*v*/*w*) of the cinnamon bark essential oils on the microbiological quality of beef. The total viable count was significantly higher in control samples on day 8th of storage. Compared to the control, in samples with 0.5% of the added essential oil, the total viable count population increased from 0.6 to 1.9 log10 CFU/g from day 4 to day 16 of storage. The highest concentration of the cinnamon bark essential oil reduced the *Enterobacteriaceae* population to 0.9 and 1.1 log CFU/g on days 12 and 16, respectively, compared to control samples. In our study, much lower concentrations (0.019, 0.038, and 0.076%) of the *Lobularia maritima* essential oil exhibited strong, antimicrobial potential in ground beef during refrigeration and storage. Further investigations that involve more detailed in vivo studies should be conducted to elucidate the antimicrobial mechanism of the tested essential oils for various applications.

#### 3.3.2. Physicochemical Analyses

##### pH

During the storage of raw beef at 4 °C for 14 days, pH values varied, depending on the addition of *LmEO* (Table 5). The initial pH values of all ground beef samples were between 5.09 and 4.91. At the end of the conservation period the pH value of the control sample reached 6.41. The pH values of the samples spiked with *LmEO*, for 14 days, showed a slight significant decrease (*p <* 0.05) indicating slightly acidic conditions. The samples treated with different concentrations of *LmEO* did not, however, differ substantially in their pHs, which were 5.59 for the samples treated with 0.019% of *LmEO*, and 5.40 for the samples treated with 0.076%. Similar results were observed when using oregano essential oil in the preservation of minced beef. Namely, the pH values of the treated samples were slightly lower of those of the control samples (*p <* 0.01) [35]. However, other authors that tested lavender and mint essential oils on ground beef meat to extend its shelf life, did not report the change in the pH of the meat stored at 4 °C and the control samples [36]. During meat storage the increase in pH is due to the progressive degradation of proteins by bacteria and certain moulds. The process is initiated by the secretion of aminases that hydrolyse NH_2_ terminal of free amino acids. The released NH_2_ is converted to ammonia (NH_3_) and ammonium ion (NH_4_^+^), which are mostly related to the production of rancid odour in spoiled meats [37]. The pH of the stored meat can increase for up to several pH units due to formation of basic compounds.

##### Protein Oxidation

Myoglobin (Mb) exists in three different forms: deoxymyoglobin (DeoMb), oxymyoglobin (OxyMb), and metmyoglobin (MetMb). The colour of fresh meat is determined by the proportion of these three pigments forms [38]. Thus, in the presence of oxygen, the purple coloured Mb can be oxygenated and converted to OxyMb, giving a bright red pigmentation. In the absence of oxygen, myoglobin oxidizes to MetMb, producing an undesirable brown colour [39]. Table 5 shows the evolution profile of MetMb in meat samples stored at 4 °C for 14 days. To ensure the sanitary quality of the meat recommended threshold for MetMb is 40% [35]. At the beginning of the storage period, the initial MetMb values in all samples were in the range between 5.55% and 8.11%.

The results represented in Table 5, show a rapid elevation (*p <* 0.05) of MetMb in untreated meat exceeding the standard quality requirements on the 14th day of storage at 4 °C (47.68%). The evolution of MetMb appeared to be slower in samples treated with the BHT standard and *LmEO*, and the values did not exceed the quality standard limits even at the 14th day of storage. The development of MetMb in samples with *LmEO* addition was slower in comparison to BHT treated meat even for the lowest tested essential oil concentration. Thus, the addition of *LmEO* proved to extend the shelf life of stored meat by at least 7 days, with respect to discoloration and protein oxidation. Soriçoban et al. [40] tested the addition of thyme and cumin essential oils to chicken meat. Similar to our findings, the MetMb values of meat samples spiked with these essential oils were found to be lower than those treated with the synthetic antioxidants BHA and BHT.

##### Lipid Oxidation

Oxidation of the lipids in meat leads to the formation of undesirable conjugated dienes (CD). At the beginning of the experiment, the contents of conjugated diets were significantly similar (*p >* 0.05) in all meat samples (Table 5). Both the standard BHT and *LmEO* reduced the formation of CDs during the entire storage period. The addition of *LmEO* once again, confirmed the positive effects on meat storage, since, in all tested concentrations, it reduced lipid peroxidation in meat, even more efficiently than the standard BHT antioxidant.

Other by-products of lipid peroxidation include thiobarbituric acid reactive substances (TBARS). A rapid and significant increase in TBARS content was observed in the control sample, reaching a maximum value of 2.48 mg MDA/kg around the 10th day of storage, exceeding the maximum permitted value (2 mg MDA/kg) [41]. However, the sample treated even with the lowest tested *LmEO* concentration (0.019%) did not exceed the maximum permitted value on day 10th. Thus, once again, the addition of *LmEO* showed to be more potent in inhibiting TBARS formation, even in comparison to standard BHT compound. It could be concluded that *LmEO* addition extended the shelf life of ground beef meat by at least 6 days compared to the control sample. The antioxidant performance of *LmEO* could have been attributed to the presence of oxygenated monoterpenes.

Previous studies reported that α- pinene and limonene exhibit significant antioxidant properties by quenching hydroxyl radicals [42]. Based on this study, essential oil of *L. maritima* appears to be excellent alternative to synthetic antioxidants that are commonly used in food preservation.

##### Sensory Analysis

Sensory properties of control meat samples were compared to those treated with the standard BHT and different concentrations of *LmEO*. The colour, odour, appearance, and overall acceptability score comparison is presented in Table 6. In samples treated with *LmEO*, colour, odour and overall acceptability were rated with higher scores for total duration of the storage period, in comparison to both control and BHT treated samples, for all tested concentrations. Odour was rated with higher scores in comparison to BHT treated samples only for *2LmEO,* and *3LmEO* samples, whereas *1LmEO* sample was comparable with the standard. Overall, the results for colour, odour, appearance, and total acceptability showed that the *3LmEO* sample, spiked with the highest essential oil concentration (0.076%) scored the highest, followed by the samples treated with *2LmEO* and *1LmEO*. All treated samples showed excellent stability of all sensory parameters up to 14 days. The changes in sensory properties reflect, in fact, the total oxidative changes of proteins and lipids. Our results were in agreement with those of Djenane et al. [36], who reported that the addition of *M. piperita* and lavender essential oils to beef meat, improved the sensory quality.

#### 3.3.3. Chemometric Analysis

To better explain the link between the *LmEO* addition to minced beef meat and main quality parameters during storage, including microbial count, protein/lipid oxidation parameters, as well as sensory parameters, a chemometric analyses applying APC and HCA approach, were applied.

##### Principal Component Analysis (PCA)

**Figure 4:**
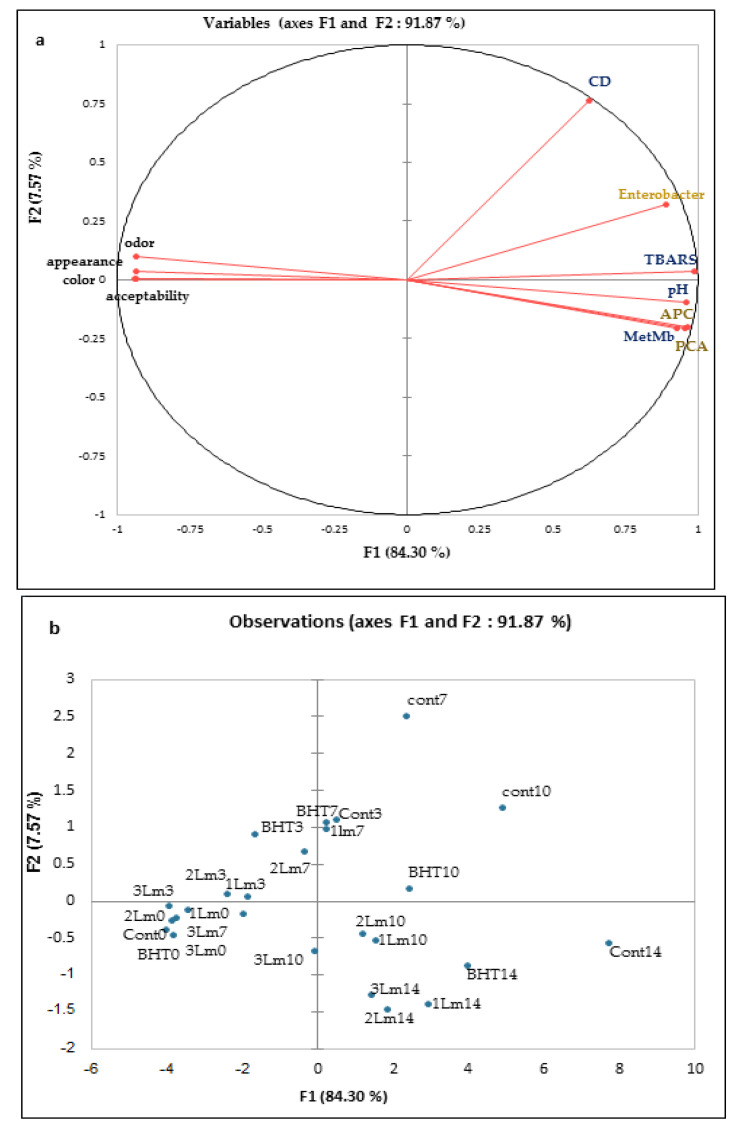
Principal component analysis (PCA) plots of microbial counts, physico-chemical parameters and sensory characteristics of meat samples at each storage time. (**a**) Variable loading plot of PCA and (**b**) Observation score plot of PCA.

PCA provided an overview of the general similarities and differences between the five samples at five storage period intervals, thus a new set of latent factors or principal components (PCs) was generated. Figure 4 depicts the PCA results for the observations presented in Table 5 and Table 6. Figure 4a showed 91.87% variance from the original data (Dim 1: 84.30%, Dim 2: 7.57%). The analysis of the data showed a strong correlation between protein oxidation (MetMb), lipid oxidation (CD and TBARS), and microbial load (PTC, APC, and Enterobacteriaceae flora), indicating that, in fact, microbial growth, was majorly responsible for these undesirable processes undergoing with meat constituents. Our results were in agreement with the research published by Guyon et al. [43] which showed that protein/lipid oxidation and microbial growth occurred at the same time and proved that lipid oxidation was the main cause of meat deterioration. Namely, meat lipids are oxidized mostly to aldehyde (malondialdehyde) interacting with other meat components (pigments, proteins, carbohydrates, and vitamins) and causing indirect oxidation of proteins [44].

The graph of factor scores (Figure 4b) shows a significant difference between 25 analysed samples. The increase in storage time led to the arrangement of the samples towards the right side of the PCA, characterized by a high concentration of lipid and proteins oxidation, and a high microbial load. Thus, with a short storage time (0–3 days), a significant and positive correlation was detected between the control, BHT, *1LmEO*, *2LmEO*, and *3LmEO* samples.

Samples treated with 0.076% of *LmEO* (*3LmEO*) were characterised with the best quality parameters, reduced micro flora proliferation, delayed chemical oxidation, and consequently the extended shelf life of raw ground beef. The oxidative stability of samples, as well as all sensory parameters, was strongly affected by the storage time.

##### Agglomerative Hierarchical Cluster Analysis (HCA) and Heat Map

**Figure 5:**
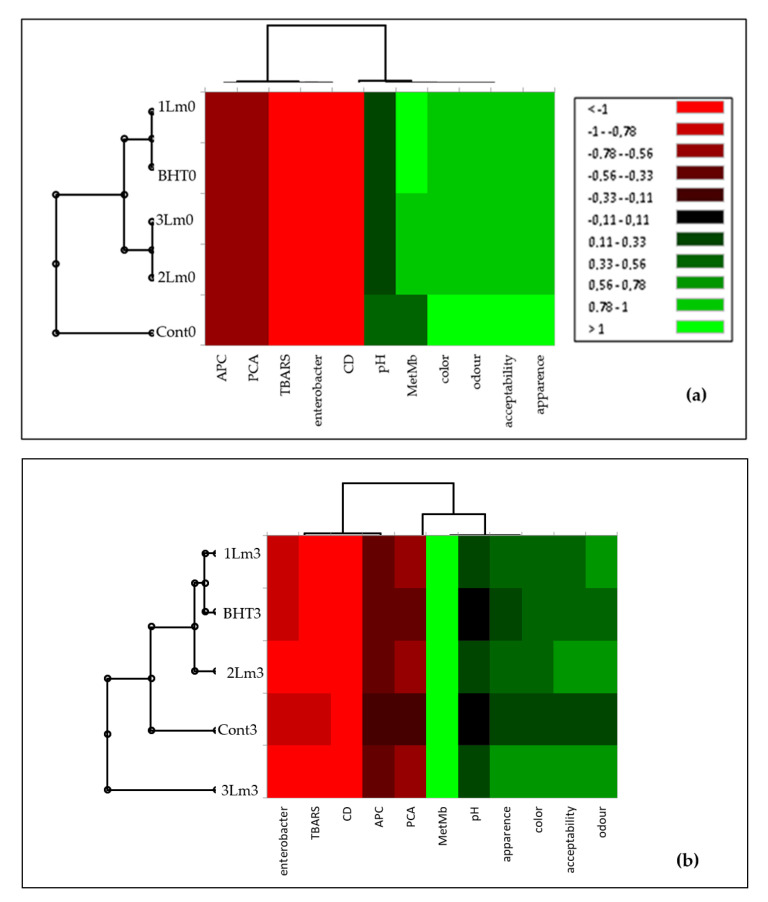

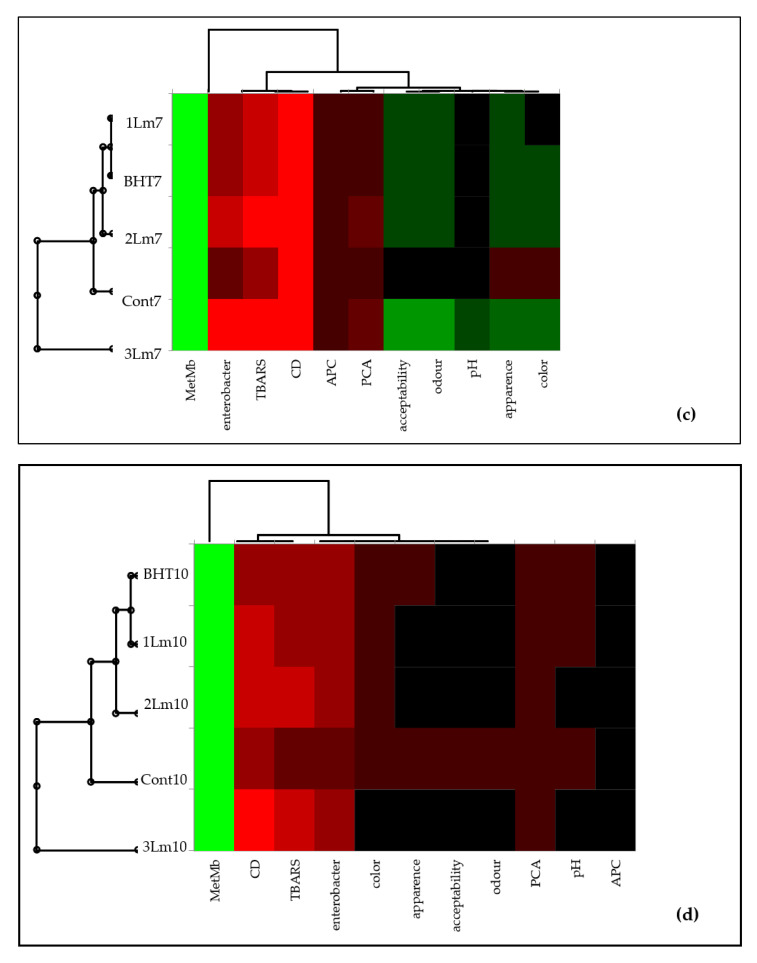

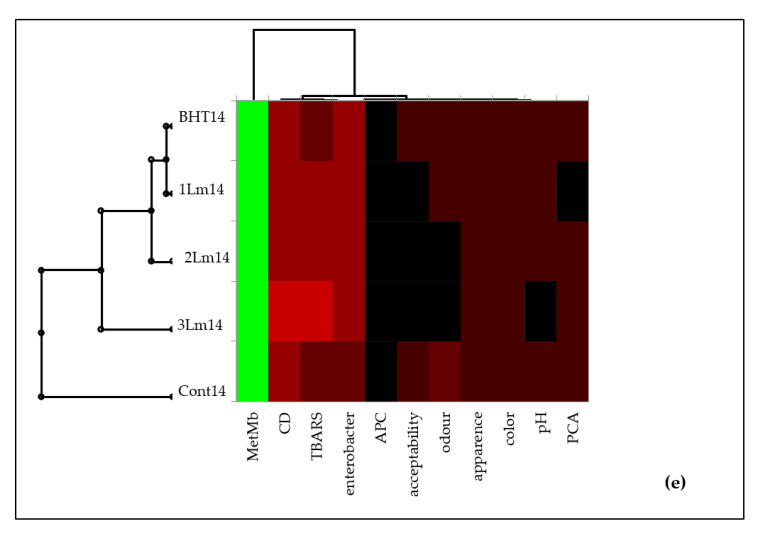
A title hierarchical cluster analysis (HCA) and heat map of physicochemical characteristics, microbial counts and sensory attributes of the control, BHT and different treated samples at each storage times: (**a**) day 0; (**b**) day 3; (**c**) day 7; (**d**) day 10 and (**e**) day 14.

To classify samples according to lipids/proteins oxidation, microbial growth, and sensory parameters during each storage time, hierarchical cluster analysis was performed, detecting the similarities between the samples (Figure 5).

At the beginning of the experiment (day 0), three distinct clusters with high similarity between the *1LmEO* and BHT samples could be noted (Figure 5a), while the control samples showed high dissimilarity in their composition. On day 3, the dendrogram demonstrated four clusters: clusters I, II, III, and IV consisting of the *1LmEO*, BHT and *2LmEO*, control and *3LmEO*, respectively (Figure 5b). This classification was based on the accumulation of TBARS, PCA and *Enterobacteriaceae*. An independence relationship was also observed between MetMb and color. As previously described by Estévez et al. [45] meat discoloration depends largely on the presence of reducing systems in the meat and lipid oxidation.

A dendrograms on day 7 (Figure 5c) and, day 10 (Figure 5d), suggest significant and linear correlation between MetMb accumulation and the increase in primary products of lipid oxidation as well as Enterobacteriaceae. Smaoui et al. [46] described the relationship between protein and lipid oxidation, proving the positive correlation. On the other hand, all sensory properties were influenced by both APC and PTC. On day 14, cluster analysis identified four clusters. The dendrogram for day 14th (Figure 5d) shows the following clusters: I (BHT-*1LmEO*), II (*2LmEO*), III (*3LmEO*), and IV (control). Relationships were noted between lipid oxidation and protein oxidation, microbial growth, and sensory changes. In addition, a relationship between CD, TBARS, and MetMb, was noted. All sensory properties were correlated with primary and secondary lipid oxidation (CD and TBARS) and protein oxidation (MetMb%) parameters, as well as microbial loads. Therefore, based on the data on microbial growth and sensory analysis, it can be concluded that chemometrics is an important tool for meat quality assessment throughout the storage.

## 4. Conclusions

The present work reports on a phytochemical and bioactivity analysis of the essential oil extracted from the Tunisian halophyte *Lobularia maritima* (*LmEO)* and reports its potential to be used as a natural meat preservative. The essential oil of *Lobularia maritima* was separated by hydrodistillation and subjected to gas chromatographic analysis. The GC MS analysis of the essential oil revealed that the major constituents were α-pinene (3.51%), benzyl alcohol (8.65%), linalool (22.43%), pulegone (3.33%), 1-phenyl butanone (7.33%), globulol (4.32%), γ-terpinene (6.15%), terpinen-4-ol (4.31%), α-terpineol (3.9%), ledol (3.59%), epi-α-cadinol (3.05%) and α-cadinol (4.91%).

The *LmEO* was further studied as a potential natural antioxidant and antimicrobial agent in minced beef meat was further studied in by in vitro and in situ analyses. Physicochemical and microbiological analyses of minced beef that was spiked with *LmEO* and stored for 14 days revealed a significant (*p <* 0.05) decrease in primary and secondary lipid oxidation and significantly (*p <* 0.05) reduced microflora proliferation. At the end of the storage period, the addition of 0.076% of *LmEO* significantly (*p <* 0.05) extended the shelf life of ground beef stored at 4 °C, inhibiting biochemical changes, and improving the overall sensory quality. Chemometric analysis, more specifically, PCA, and HCA, provided useful insights correlating oxidation and microbiological processes to sensory properties of the stored minced beef meat. The present investigation represents the first report on the use of the essential oil of *Lobularia maritima* in beef meat preservation and evaluation of the spiked beef meat samples by multivariate exploratory techniques. This study showed that *LmEO* can be an encouraging natural preservative for the meat industry.

## Figures and Tables

**Table 1:** The chemical composition of *Lobularia maritima* essential oil.

No.	Compound ^a^	KI ^b^	% ^c^
1	Furfural	800	0.18
2	α Thujene	883	0.12
3	α-Pinene	938	3.51
4	Sabinene	976	2.13
5	Myrcene	947	0.15
6	α-Phellandrene	1003	0.37
7	δ-3-Carene	1016	0.15
8	Benzyl alcohol	1040	8.65
9	γ -Terpinene	1052	6.15
10	Acetophenone	1065	0.15
11	Z-linalool oxide (furanoid)	1074	0.16
12	Linalool	1082	22.43
13	(E)-2-Undecene	1105	1.6
14	Camphor	1115	2.6
15	1-Terpineol	1143	5.6
16	Terpinen-4-ol	1175	4.31
17	α-Terpineol	1176	3.9
18	Pulegone	1238	3.33
19	δ-Elemene	1338	0.22
20	Isoledene	1376	0.37
21	α-Copaene	1379	0.22
22	β-Bournonen	1380	0.26
23	β-Cubebene	1384	0.24
24	α-Gurjunene	1406	0.22
25	1-Phenyl butanone	1425	7.33
26	α-Humulene	1455	0.15
27	Germacrene D	1462	0.42
28	β-Selinene	1484	0.24
29	β-Sesquiphellandrene	1501	0.17
30	Germacrene B	1535	0.34
31	Ledol	1561	5.59
32	Germacrene D-4-ol	1573	0.12
33	Spathulenol	1576	0.47
34	Globulol	1590	6.32
35	1.10-di-epi-Cubenol	1627	1.67
36	10-epi-γ-Eudesmol	1635	0.27
37	Epi-α-Cadinol	1643	3.05
38	α-Cadinol	1672	4.91
39	Z-methyl epijasmonate	1675	0.14
40	Z-α-bisabolene epoxide	1680	0.54
Monoterpene Hydrocarbon Oxygenated Monoterpenes	-	- -	24.13 74.40
Total (%)			98.53

^a^ Identification of compounds based on GCMS Wiley 7.0 version library and National Institute of Standards and Technology 05 MS (NIST) library data; ^b^ KI: Kovats Indices on HP-5MS Capillary Column in reference to C_10_-C_22_ n-alkanes under same conditions; ^c^ %: Percentages are the means of two runs and were obtained from electronic integration measurements using a selective mass detector.

**Table 2:** Antibacterial activity of *LmEO* expressed as inhibition zones.

Bacterial Strain	Inhibition Zone Diameter (mm)				
	*LmEO*	1/2	1/4	1/8	STR
Gram-positive					
*Bacillus cereus* ATCC 14579 *Staphylococcus aureus* ATCC 25923	28.6 ± 0.8 22.5 ± 0.5	22.5 ± 0.6 19 ± 0.5	19 ± 0.01 16 ± 0.5	18 ± 0.5 12 ± 0.1	23.3 ± 0.0 14.3 ± 0.3
*Enterococcus faecalis* ATCC 29212 *Micrococcus luteus* ATCC 1880	27.3 ± 0.3 25.6 ± 0.3	24.5 ± 0.1 19.5 ± 0_._0	23 ± 0.3 18 ± 0.3	18 ± 0.5 12 ± 0.5	26.6 ± 0.6 13.6 ± 0.3
*Listeria monocytogenes* ATCC 1911	26.6 ± 0.1	23.5 ± 0.5	23 ± 0.5	21 ± 0.4	25.3 ± 0.5
Gram-negative					
*Pseudomonas aeruginosa* ATCC 9027	27.6 ± 0.3	23.5 ± 0.5	21 ± 0.5	18 ± 0.5	21.6 ± 0.5
*Escherichia coli* ATCC 25922	29 ± 0.6	22.5 ± 0.5	21 ± 0.5	18 ± 0.01	19.6 ± 0.3
*Salmonella enterica* ATCC 43972	33 ± 0.3	26 ± 0.01	23 ± 0_._5	17.5 ± 0.5	27.3 ± 0.3

*LmEO*: raw oil at concentration 1.8 mg/well; 1/2: *LmEO* = 0.9 mg/well; 1/4: *LmEO* = 0.45 mg/well; 1/8: *LmEO* = 0.225 mg/well; STR: reference antibiotic Streptomycin at 20 μg/well. The values are expressed as mean ± SEM (*n* = 3).

**Table 3:** Minimum Inhibitory Concentrations (MIC) and Minimum Bactericidal Concentrations (MBC) of *LmEO*.

Bacterial Strain	MIC (µg/mL)	MBC (µg/mL)	MBC/MIC	Antibacterial Activity
Gram-positive				
*Bacillus cereus* ATCC 14579 *Staphylococcus aureus* ATCC 25923	55 ± 0.00 32 ± 0.00	65 ± 0.00 250 ± 0.05	1 7	Bactericidal Bacteriostatic
*Enterococcus faecalis* ATCC 29212 *Micrococcus luteus* ATCC 1880	65 ± 0.02 25 ± 0.01	410 ± 0.15 200 ± 0.00	6 8	Bacteriostatic Bacteriostatic
*Listeria monocytogenes* ATCC 1911	19 ± 0.00	102 ± 0.02	5	Bacteriostatic
Gram-negative				
*Pseudomonas aeruginosa* ATCC 9027	25 ± 0.00	25 ± 0.00	1	Bactericidal
*Escherichia coli ATCC* 25922	32 ± 0.31	260 ± 0.18	8	Bacteriostatic
*Salmonella enterica* ATCC 43972	29 ± 0.01	32 ± 0.00	1	Bactericidal

**Table 4:** The effects of *LmEO* on the aerobic plate count (APC), psychrotrophic count (PTC), and *Enterobacteriaceae* count of raw minced meat beef stored at 4 °C.

Days of Storage at 4 °C					
	0	3	7	10	14
APC					
C	2.29 * ± 0.35 ^aA^	3.56 ± 0.07 ^aB^	4.69 ± 0.07 ^dC^	6.65 ± 0.03 ^dB^	7.57 ± 0.14 ^dE^
BHT *1LmEO* *2LmEO*	2.25 ± 0.17 ^aA^ 2.29 ± 0.21 ^aA^ 2.27 ± 0.01 ^aA^	3.42 ± 0.1 ^aB^ 3.41 ± 0.35 ^aB^ 3.28 ± 0.28 ^aB^	4.38 ± 0.21 ^cC^ 4.14 ± 0.18 ^cC^ 4.09 ± 0.28 ^aC^	6.07 ± 0.14 ^cB^ 6.27 ± 0.28 ^cB^ 5.99 ± 0.28 ^bB^	6.72 ± 0.07 ^bcC^ 6.60 ± 0.28 ^cC^ 6.32 ± 0.35 ^bC^
*3LmEO*	2.17 ± 0.14 ^aA^	3.12 ± 0.1 ^aB^	3.94 ± 0.21 ^aC^	4.87 ± 0.01 ^aC^	5.91 ± 0.01 ^aB^
PTC					
C	2.01 ± 0.02 ^aA^	2.68 ± 0.08 ^dB^	4.00 ± 0.05 ^dC^	5.07 ± 0.02 ^eD^	6.33 ± 0.05 ^dD^
BHT *1LmEO* *2LmEO*	2.03 ± 0.23 ^aA^ 1.96 ± 0.05 ^aA^ 1.93 ± 0.54 ^aA^	2.97 ± 0.2 ^cB^ 2.13 ± 0.08 ^bB^ 2.18 ± 0.82 ^bB^	3.77 ± 0.86 ^cBC^ 3.57 ± 0.01 ^bBC^ 3.48 ± 0.28 ^aC^	4.72 ± 0.08 ^dCD^ 4.64 ± 0.05 ^cD^ 4.53 ± 0.28 ^bD^	6.04 ± 0.01 ^cD^ 5.41 ± 0.26 ^bD^ 5.15 ± 0.02 ^aE^
*3LmEO*	1.94 ± 0.02 ^aA^	1.95 ± 0.02 ^aB^	3.01 ± 0.05 ^aC^	4.11 ± 0.08 ^aC^	4.79 ± 0.02 ^aE^
*Enterobacteriaceae* count					
C	<1	2.36 ± 0.02 ^dA^	2.68 ± 0.05 ^eA^	3.12 ± 0.25 ^bAB^	3.47 ± 0.13 ^cB^
BHT *1LmEO* *2LmEO*	<1 <1 <1	1.66 ± 0.08 ^cA^ 1.49 ± 0.14 ^bA^ 1.40 ± 0.05 ^bA^	1.97 ± 0.14 ^dAB^ 1.85 ± 0.08 ^cB^ 1.67 ± 0.05 ^bB^	2.27 ± 0.21 ^bAC^ 1.95 ± 0.02 ^aC^ 1.82 ± 0.05 ^aC^	2.48 ± 0.08 ^bC^ 2.00 ± 0.05 ^aD^ 1.96 ± 0.05 ^aD^
*3LmEO*	<1	<1	1.43 ± 0.02 ^aA^	1.64 ± 0.08 ^aAB^	1.78 ± 0.23 ^aB^

* log10 UFC/g of meat ± SEM of three replicates; Values with a different letter (a–e) of the same storage day are significantly different (*p <* 0.05); Values with a different letter (A–E) of the same concentration are significantly different.

**Table 5:** The effects of *LmEO* on pH, MetMb, CD and TBARS contents in raw minced meat.

Days of Storage at 4 °C						
		0	3	7	10	14
pH	C	5.09 ± 0.07 ^aA^	5.27 ± 0.05 ^bAB^	5.5 ± 0.03 ^cB^	5.90 ± 0.03 ^cC^	6.41 ± 0.015 ^cD^
	BHT *1LmEO* *2LmEO*	5.08 ± 0.02 ^aA^ 4.96 ± 0.03 ^aA^ 4.91 ± 0.03 ^aA^	5.22 ± 0.04 ^abA^ 5.18 ± 0.02 ^abB^ 5.14 ± 0.21 ^abB^	5.30 ± 0.09 ^bAB^ 5.24 ± 0.01a ^bB^ 5.21 ± 0.02 ^aB^	4.53 ± 0.09 ^bB^ 5.52 ± 0.05 ^bC^ 5.45 ± 0.035 ^abC^	5.88 ± 0.02 ^bC^ 5.59 ± 0.01 ^aC^ 5.59 ± 0.07 ^aC^
	*3LmEO*	4.93 ± 0.03 ^aA^	5.05 ± 0.05 ^aB^	5.15 ± 0.01 ^aBC^	5.22 ± 0.01 ^aC^	5.40 ± 0.03 ^aD^
TBARS (mg MDA-eq/kg)	C	0.35 ± 0.07 ^bA^	1.56 ± 0.01 ^cB^	1.97 ± 0.17 ^bBC^	2.48 ± 0.21 ^cCD^	3.07 ± 0.14 ^cD^
	BHT *1LmEO* *2LmEO*	0.06 ± 0.02 ^aA^ 0.17 ± 0.01 ^aA^ 0.08 ± 0.01 ^aA^	0.86 ± 0.03 ^dB^ 0.76 ± 0.02 ^cB^ 0.57 ± 0.01 ^bB^	1.17 ± 0.11 ^abB^ 1.30 ± 0.25 ^abB^ 1.13 ± 0.01 ^abB^	2.19 ± 0.22 ^bcC^ 1.70 ± 0.21 ^abB^ 1.30 ± 0.15 ^abB^	2.47 ± 0.17 ^acC^ 2.11 ± 0.13 ^abB^ 1.68 ± 0.13 ^abB^
	*3LmEO*	0.04 ± 0.13 ^aA^	0.24 ± 0.03 ^aB^	0.63 ± 0.19 ^aAB^	1.12 ± 0.06 ^aBC^	1.47 ± 0.15 ^ac^
CD (μmole/mg of meat)	C	0.69 ± 0.42 ^aA^	0.78 ± 0.01 ^aAB^	0.86 ± 0.08 ^bB^	0.85 ± 0.04 ^aB^	0.79 ± 0.02 ^cC^
	BHT *1LmEO* *2LmEO*	0.68 ± 0.21 ^aA^ 0.68 ± 0.01 ^aA^ 0.67 ± 0.03 ^aA^	0.75 ± 0.5 ^aAB^ 0.73 ± 0.2 ^aA^ 0.73 ± 0.01 ^aB^	0.80 ± 0.01 ^aA^ 0.83 ± 0.06 ^bB^ 0.76 ± 0.1 ^abA^	0.79 ± 0.07 ^abA^ 0.73 ± 0.95^05 abA^ 0.74 ± 0.01 ^abAB^	0.75 ± 0.04 ^bA^ 0.73 ± 0.05 ^bA^ 0.69 ± 0.04 ^aAB^
	*3LmEO*	0.69 ± 0.05 ^aAB^	0.71 ± 0.01 ^aB^	0.73 ± 0.01 ^aA^	0.69 ± 0.01 ^aAB^	0.68 ± 0.05 ^aAB^
MetMb (%)	C	5.55 ± 0.42 ^aA^	15.3 ± 0.41 ^dB^	18.43 ± 0.56 ^dC^	32.85 ± 0.05 ^cD^	47.68 ± 0.44 ^eE^
	BHT *1LmEO* *2LmEO*	8.11 ± 0.21 ^bA^ 7.89 ± 0.1 ^bA^ 6.89 ± 0.03 ^abA^	14.4 ± 0.52 ^cdB^ 12.78 ± 0.38 ^bcB^ 11.41 ± 0.29 ^abB^	16.62 ± 0.01 ^cdB^ 16.30 ± 0.2 ^cC^ 13.87 ± 0.45 ^bC^	29.27 ± 0.56 ^bcC^ 25.70 ± 1.2 ^bD^ 21.68 ± 0.75 ^aD^	32.6 ± 0.24 ^bD^ 30.44 ± 0.09 ^cE^ 28.46 ± 0.05 ^bE^
	*3LmEO*	7.07 ± 0.31 ^bA^	9.80 ± 0.05 ^aB^	11.41 ± 0.06 ^aB^	18.43 ± 0.58 ^aC^	21.33 ± 0.54 ^aD^

± SEM of three replicates; Values with a different letter (a–e) of the same storage day are significantly different (*p <* 0.05); Values with a different letter (A–E) of the same concentration are significantly different.

**Table 6:** The influence of *LmEO* addition on sensory properties of raw minced beef meat.

Days of Storage at 4 °C						
		0	3	7	10	14
colour	C	6.8 ± 0.25 ^aD^	5.6 ± 0.16 ^aC^	5 ± 0.25 ^aBC^	4.33 ± 0.42 ^aAB^	3.8 ± 0.30 ^aA^
	BHT *1LmEO* *2LmEO*	7.2 ± 0.14 ^aD^ 6.5 ± 0.33 ^aB^ 7.2 ± 0.32 ^aC^	6.5 ± 0.29 ^abCD^ 6.1 ± 0.23 ^abAB^ 6.4 ± 0.24 ^abAB^	6 ± 0.25 ^cBC^ 5.3 ± 0.42 ^abAB^ 5.8 ± 0.30 ^abAB^	5 ± 0.36 ^abAB^ 5.2 ± 0.36 ^abA^ 5.16 ± 0.30 ^abA^	4.5 ± 0.22 ^abA^ 4.8 ± 0.30 ^abA^ 4.6 ± 0.21 ^abA^
	*3LmEO*	7.3 ± 0.16 ^aC^	6.6 ± 0.16 ^cBC^	6.4 ± 0.25 ^cAB^	5.5 ± 0.34 ^cA^	5.16 ± 0.30 ^cA^
Odour	C	7.03 ± 0.23 ^aD^	6.3 ± 0.17 ^aCD^	5.5 ± 0.22 ^aBC^	4.6 ± 0.21 ^aB^	3.3 ± 0.21 ^aA^
	BHT *1LmEO* *2LmEO*	6.8 ± 0.20 ^aC^ 6.8 ± 0.2 ^aC^ 7 ± 0.16 ^aB^	6.6 ± 0.03 ^aBC^ 6.5 ± 0.30 ^aBC^ 6.7 ± 0.34 ^aB^	6.1 ± 0.16 ^abBC^ 5.8 ± 0.16 ^abBC^ 6.3 ± 0.21 ^abAB^	5.6 ± 0.21 ^abAB^ 5.6 ± 0.33 ^abAB^ 6.4 ± 0.25 ^cAB^	5.4 ± 0.25 ^bA^ 5.1 ± 0.30 ^bA^ 5.6 ± 0.21 ^bA^
	*3LmEO*	7.2 ± 0.32 ^aA^	7.1 ± 0.27 ^aA^	6.6 ± 0.66 ^cA^	6.3 ± 0.33 ^cA^	6.0 ± 0.36 ^bA^
Appearance	C	6.5 ± 0.33 ^aC^	5.8 ± 0.26 ^aBC^	5 ± 0.25 ^aB^	4.66 ± 0.21 ^aAB^	3.6 ± 0.33 ^aA^
	BHT *1LmEO* *2LmEO*	6.6 ± 0.28 ^aC^ 7.1 ± 0.2 ^aC^ 7.3 ± 0.2 ^aC^	6 ± 0.37 ^aBC^ 6.1 ± 0.2 ^aB^ 6.4 ± 0.44 ^aBC^	5.5 ± 0.22 ^abAB^ 5.6 ± 0.21 ^abAB^ 6 ± 0.36 ^abBC^	5 ± 0.25 ^abAB^ 5.5 ± 0.22 ^abAB^ 5.6 ± 0.21 ^cAB^	4.6 ± 0.21 ^abA^ 4.8 ± 0.30 ^cA^ 5.0 ± 0.25 ^cA^
	*3LmEO*	7.2 ± 0.14 ^aC^	6.6 ± 0.28 ^aBC^	6.3 ± 0.33 ^cBC^	5.8 ± 0.16 ^cAB^	5.8 ± 0.16 ^cA^
Overall acceptability	C	6.5 ± 0.17 ^aB^	6 ± 0.26 ^aB^	5.8 ± 0.16 ^aB^	4.51 ± 0.34 ^aA^	3.6 ± 0.15 ^aA^
	BHT *1LmEO* *2LmEO*	7.2 ± 0.22 ^aC^ 6.8 ± 0.2 ^aB^ 7.1 ± 0.2 ^aB^	6.7 ± 0.22 ^abBC^ 6.4 ± 0.24 ^abAB^ 6.8 ± 0.26 ^abAB^	6 ± 0.36 ^abAB^ 6 ± 0.36 ^abAB^ 6.3 ± 0.25 ^abAB^	5.6 ± 0.42 ^abAB^ 5.8 ± 0.16 ^abA^ 6.0 ± 0.47 ^cAB^	4.8 ± 0.3 ^bA^ 5 ± 0.22 ^bA^ 5.6 ± 0.21 ^bA^
	*3LmEO*	7.2 ± 0.22 ^aB^	7 ± 0.23 ^cB^	6.6 ± 0.21 ^cAB^	6.3 ± 0.33 ^cAB^	5.8 ± 0.30 ^bA^

± SEM of three replicates; Values with a different letter (a–c) of the same storage day are significantly different (*p* < 0.05); values with a different letter (A–D) of the same concentration are significantly different.

## Data Availability

The data presented in this study are available on request from Anis Ben Hsouna.
